# Slow but Evident Recovery from Neocortical Dysfunction and Cognitive Impairment in a Series of Chronic COVID-19 Patients

**DOI:** 10.2967/jnumed.121.262128

**Published:** 2021-03-31

**Authors:** Ganna Blazhenets, Nils Schroeter, Tobias Bormann, Johannes Thurow, Dirk Wagner, Lars Frings, Cornelius Weiller, Philipp T. Meyer, Andrea Dressing, Jonas A. Hosp

**Affiliations:** 1 Department of Nuclear Medicine, Faculty of Medicine, Medical Center–University of Freiburg, University of Freiburg, Freiburg, Germany;; 2 Department of Neurology and Clinical Neuroscience, Faculty of Medicine, Medical Center–University of Freiburg, University of Freiburg, Freiburg, Germany;; 3 Department of Internal Medicine, Faculty of Medicine, Medical Center–University of Freiburg, University of Freiburg, Freiburg, Germany; and; 4 Freiburg Brain Imaging Center, Faculty of Medicine, Medical Center–University of Freiburg, University of Freiburg, Freiburg, Germany

**Keywords:** COVID-19, cognition, neurology, 18F-FDG PET, Montreal Cognitive Assessment

## Abstract

Cognitive impairment is a frequent complaint in coronavirus disease 2019 (COVID-19) and can be related to cortical hypometabolism on ^18^F-FDG PET at the subacute stage. However, it is unclear if these changes are reversible. **Methods:** We prospectively assessed the Montreal Cognitive Assessment scores and ^18^F-FDG PET scans of 8 COVID-19 patients at the subacute stage (once no longer infectious) and the chronic stage (˜6 mo after symptom onset). The expression of the previously established COVID-19–related covariance pattern was analyzed at both stages to examine the time course of post–COVID-19 cognitive impairment. For further validation, we also conducted a conventional group analysis. **Results:** Follow-up ^18^F-FDG PET revealed that there was a significant reduction in the initial frontoparietal and, to a lesser extent, temporal hypometabolism and that this reduction was accompanied by a significant improvement in cognition. The expression of the previously established COVID-19–related pattern was significantly lower at follow-up and correlated inversely with Montreal Cognitive Assessment performance. However, both ^18^F-FDG PET and cognitive assessment suggest a residual impairment. **Conclusion:** Although a significant recovery of regional neuronal function and cognition can be clearly stated, residuals are still measurable in some patients 6 mo after manifestation of COVID-19. Given the current pandemic situation and tremendous uncertainty concerning the long-term effects of COVID-19, the present study provides novel insights of the highest medical and socioeconomic relevance.

## Visual Abstract

**Figure:**
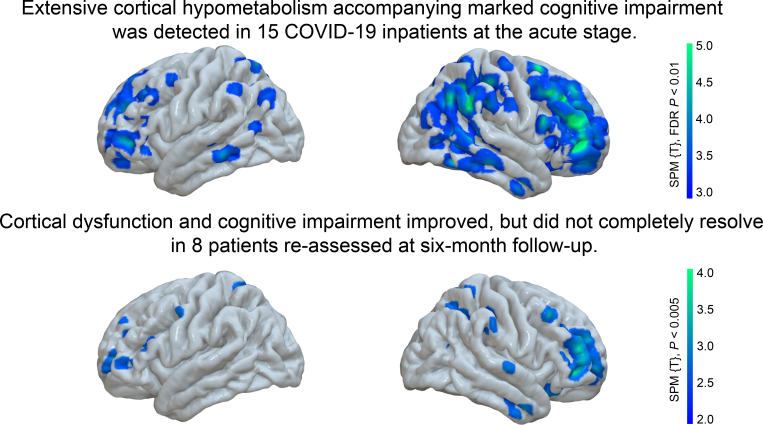

As the severe acute respiratory syndrome coronavirus 2 (SARS-CoV-2) pandemic proceeds, neurocognitive long-term consequences are frequently observed (*1*): follow-up investigations of coronavirus disease 2019 (COVID-19) patients 2–4 mo after symptom onset report, among others, impaired memory (20%–34%) (*2,3*), disturbed concentration (20%–40%) (*2,3*), and cognitive problems (36%) (*4*). These cognitive deficits in the chronic stage are now frequently referred to as long COVID-19 or as post–COVID-19 syndrome. Recently, we described impairment of frontoparietal cognitive functions accompanied by frontoparietally dominant cortical hypometabolism on ^18^F-FDG PET (as an established marker of neuronal function) in a relevant subset of subacute COVID-19 patients initially requiring inpatient treatment for nonneurologic complications (*5*). By comparing the ^18^F-FDG PET scans of those subacute COVID-19 inpatients with a control sample using voxelwise principal-components analysis, we established a COVID-19–related spatial covariance pattern, the expression of which tightly correlated with performance in the Montreal Cognitive Assessment (MoCA) (*6*). Frontal and, to a lesser extent, temporoparietal cortical hypometabolism, which improved during follow-up at 1 and 6 mo, was also confirmed as a major finding in the acute phase of COVID-19–related encephalopathy by a recent study of Kas et al. (*7*). Deviating from the aforementioned results, Guedj et al. (*8*) reported a profile of hypometabolism in limbic or paralimbic regions extending to the brain stem and cerebellum in patients with long COVID-19 examined at about 3 mo after symptom onset. Against this background, we investigated whether the frontoparietal hypometabolism might be a biologic fingerprint of post–COVID-19 syndrome neurocognitive impairments. We reassessed ^18^F-FDG PET and MoCA performance in 8 patients presenting for follow-up during the chronic stage approximately 6 mo after symptom onset.

## MATERIALS AND METHODS

### Standard Protocol Approvals, Registrations, and Patient Consents

The patients were part of a prospective monocentric register (Neuro-COVID-19). The local ethics committee approved this study (EK 211/20), and all subjects gave written informed consent in accordance with the Declaration of Helsinki.

### Study Design, Participants, and Assessment of Cognitive Functions

The register enrolled patients who had reverse transcription polymerase chain reaction–confirmed SARS-CoV-2 infection, whose case of COVID-19 developed least 1 novel neurologic symptom, and who required inpatient treatment in the Department of Internal Medicine of the University Hospital Freiburg between April 20, 2020, and June 10, 2020 (details are provided by Hosp et al. (*5*)). During the acute stage, 31 patients had been assessed for impaired cognitive functions with the MoCA (German, version 7.1) (*6*). Seventeen of these patients had undergone an ^18^F-FDG PET examination at the subacute stage. Of those, 8 patients underwent a second examination with ^18^F-FDG PET and the MoCA (German, alternative version 7.2) at the chronic stage of the disease and were included in this study. Eight patients refused further investigations (no more self-perceived complaints: *n* = 6; long traveling distance: *n* = 1; bad physical condition: *n* = 1), and 1 patient died.

Of note, the present population does not represent long COVID-19 (typically defined by long-lasting, not exclusively neurologic complaints at least 4–12 wk after symptom onset (*9*)) since enrollment into this prospective study was based on at least 1 new neurologic symptom at the subacute stage (on average, 29 ± 15 d after COVID-19 symptom onset) and then patients were followed up to investigate the reversibility of symptoms. In fact, 4 of 8 patients (50%) did not have any more self-reported cognitive deficits at the time of the second examination (Table 1).

**TABLE 1:** Patient Demographic and Basic Clinical Characteristics and Results of MoCA

Characteristic	Data
Demographic data	
Age (y)	66.00 (14.23) (39–89)
Sex	
Male	6 (75%)
Female	2 (25%)
Years of education	12.63 (2.74) (9–18)
Delay after symptom onset, first exam (d)	28.50 (14.63) (13–61)
Delay after symptom onset, second exam (d)	160.13 (46.79) (113–233)
Delay from first PET to second PET (d)	123.13 (39.61) (87–196)
Self-reported persistent cognitive deficits	4 (50%)
Characteristics of initial inpatient treatment	
Reduced general condition	2 (25%)
Bacterial pulmonary superinfection	2 (25%)
Kidney failure	3 (37.5%)
Ischemic stroke	1 (12.5%)
Required intensive-care-unit treatment	2 (25%)
MoCA corrected for years of education	
MoCA, first exam	19.13 (4.51) (13–25)
MoCA, second exam	23.38 (3.60) (17–28)
Δ MoCA (from second exam to first exam)	4.25 (4.20)
MoCA domain scores	
Orientation (maximum, 6)	6.00 (0.00) (6)
Attention (maximum, 6)	5.13 (0.83) (4–6)
Language (maximum, 6)	3.88 (1.96) (1–6)
Visuoconstructive functions (maximum, 4)	3.13 (0.99) (2–4)
Executive functions (maximum, 4)	2.50 (1.07) (1–4)
Memory (maximum, 5)	2.25 (2.12) (0–5)

Qualitative data are number and percentage; continuous data are mean, SD, and range.

The manifestation of cognitive deficits was further described by domain scores on the MoCA test as suggested by Nasreddine et al. (*6*) based on single-item scores (orientation: spatial and temporal orientation; attention: digit span, letter A tapping, subtraction; executive: Trail Making Test, abstraction, word fluency; visuoconstructive: cube copying, clock drawing; language: naming, sentence repetition; memory: delayed word recall). The global MoCA test score was corrected for years of education (+1 point if ≤12 y of education). Domain scores were not adjusted for years of education.

### ^18^F-FDG PET Imaging

PET emission data were acquired for 10 min on a fully digital Vereos PET/CT scanner (Philips Healthcare) 50 min after injection of 213 ± 9 MBq of ^18^F-FDG. ^18^F-FDG PET scans were spatially normalized to an in-house ^18^F-FDG PET template in Montreal Neurologic Institute space, followed by smoothing with an isotropic gaussian kernel of 10 mm in full width at half maximum. The topographic profile rating algorithm (*10*) of the ScAnVP software (The Fenstein Institute for Medical Research) was used to derive each individual’s pattern expression score (PES) for the previously established COVID-19–related spatial covariance pattern (*5*). For additional conventional analysis, a paired *t* test between the ^18^F-FDG PET scans at the subacute and chronic stages was performed after proportional scaling of individual voxelwise ^18^F-FDG uptake to white matter (given the obvious involvement of gray matter shown in the previous study (*5*)). Voxelwise 2-sample *t* testing was also applied to the ^18^F-FDG PET scans of COVID-19 patients at the chronic stage compared with the control cohort (*n* = 45 age-matched control patients in whom a somatic central nervous system disease was carefully excluded (*5*)) in order to explore whether hypometabolism still remains at the chronic stage of disease. All processing steps were implemented with an in-house pipeline in MATLAB (The MathWorks, Inc.) and Statistical Parametric Mapping, version 12 (SPM12), software (www.fil.ion.ac.uk/spm).

### Statistical Analysis

The significance of differences between subacute and chronic stages for MoCA test scores and the PES for the COVID-19–related covariance pattern was assessed with paired *t* tests. The Student 1-tailed *t* test was applied to determine whether the PES in COVID-19 patients at the subacute stage was still significantly higher than in the control cohort. Cohen d was calculated for all pairwise comparisons. The strength of the relationship between the MoCA and the PES for the COVID-19–related covariance pattern was estimated within the stages by linear regression and across stages with a repeated-measures correlation test (*11*). Statistical analyses were performed using R (https://www.R-project.org/).

### Data Availability

The data generated and analyzed during the current study are not publicly available but could possibly be provided by the corresponding author on reasonable request and on approval by the local ethics committee.

## RESULTS

**FIGURE 1.**
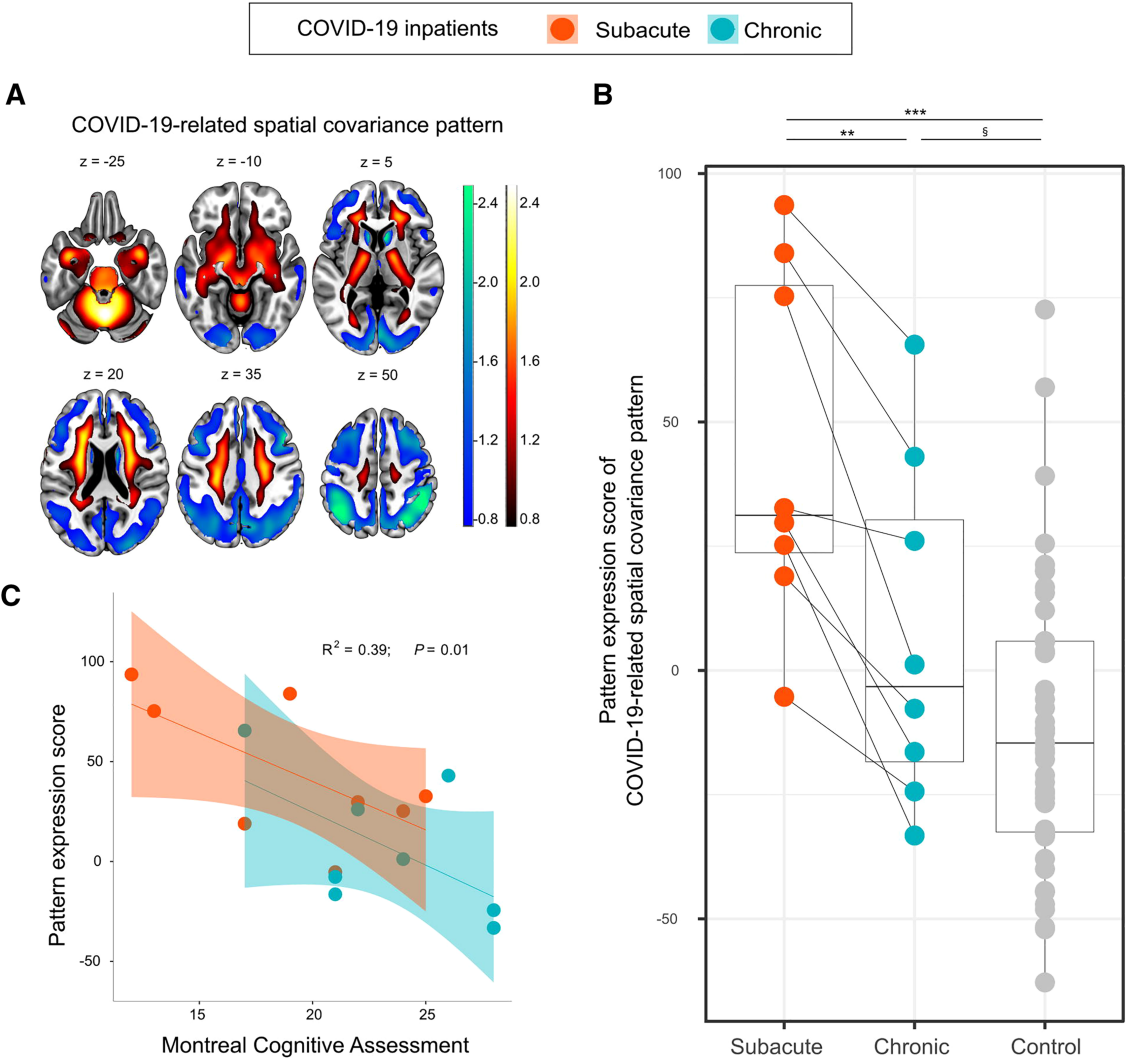
Expression of COVID-19–related spatial covariance pattern. (A) COVID-19–related spatial covariance pattern of cerebral glucose metabolism established in Hosp et al. (*5*) overlaid onto MRI template. Regions with negative weights are coded in cool colors, and regions with positive weights are coded in warm colors (neurologic orientation, i.e., left image side corresponds to patient’s left body side; numbers denote axial [*z*] position in mm). (B) Graph showing that PES for COVID-19–related spatial covariance pattern is lower at chronic stage than at subacute stage but is still, at trend level, higher than in control cohort. Box plots, as well as individual values for COVID-19 patients (colored) and control cohort (gray), are displayed. Repeated measures for each patient are connected by line. ***P < 0.001 (2-sample *t* test (*5*)). **P < 0.005 (2-tailed paired *t* test). ^§^*P* = 0.06 (1-tailed 2-sample *t* test). (C) Association between PES and MoCA score adjusted for years of education. Each dot represents individual patient’s data; shaded areas correspond to fit of linear regression (95% CI) for each disease stage separately (*P* = 0.07 and 0.12 for subacute and chronic stages, respectively). Repeated-measures *R*^2^ and *P* value represent correlation between variables with both stages pooled.

Demographics and patient characteristics are listed in Table 1. The 8 patients presenting for a follow-up examination were not distinct from the rest of the cohort at the baseline examination (PES for COVID-19–related covariance pattern on ^18^F-FDG PET, MoCA, and age did not significantly differ between groups; all *P* > 0.1). MoCA performance significantly improved over time from a mean (±SD) global score of 19.1 ± 4.5 (maximum, 30 points) at the subacute stage to 23.4 ± 3.6 at the chronic stage (d = 0.97, *P* = 0.03; Table 1)—a score that is, however, still below the frequently used cutoff for detection of cognitive impairment (<26/30). Five of 8 patients still were below this threshold (*6*). MoCA domain scores showed that orientation and attention were almost unimpaired at the chronic stage but revealed persistent deficits in visuoconstructive and executive functions and, especially, memory (Table 1). As previously shown (*5*), the PES for the COVID-19–related pattern (Fig. 1A) in the patients with subacute COVID-19 was significantly higher than that in the control cohort (44.3 vs. −11.3; d = 1.84, *P* = 6 × 10^−6^). COVID-19 patients had a significantly lower mean PES at the chronic than at the subacute stage (6.8 ± 32.6 vs. 44.3 ± 33.1; d = 1.06, *P* = 0.002) although still, at trend level, higher than in the control cohort (6.8 vs. −11.3; d = 0.60, *P* = 0.06) (Fig. 1B). Exploratory correlation analysis revealed a significant relationship between cognitive assessment (MoCA global score adjusted for years of education) and PET (*R*^2^ = 0.39, *P* = 0.01; i.e., a lower PES was associated with better cognitive performance) over the subacute and chronic stages (Fig. 1C). Moreover, changes in cognition (MoCA) seem to be associated with changes in PES but failed to attain statistical significance in the present small sample (*r* = −0.54, *P* = 0.16, *n* = 8). No significant correlation was found between changes in PES and MoCA on the one side and time to follow-up examinations on the other.

**FIGURE 2.**
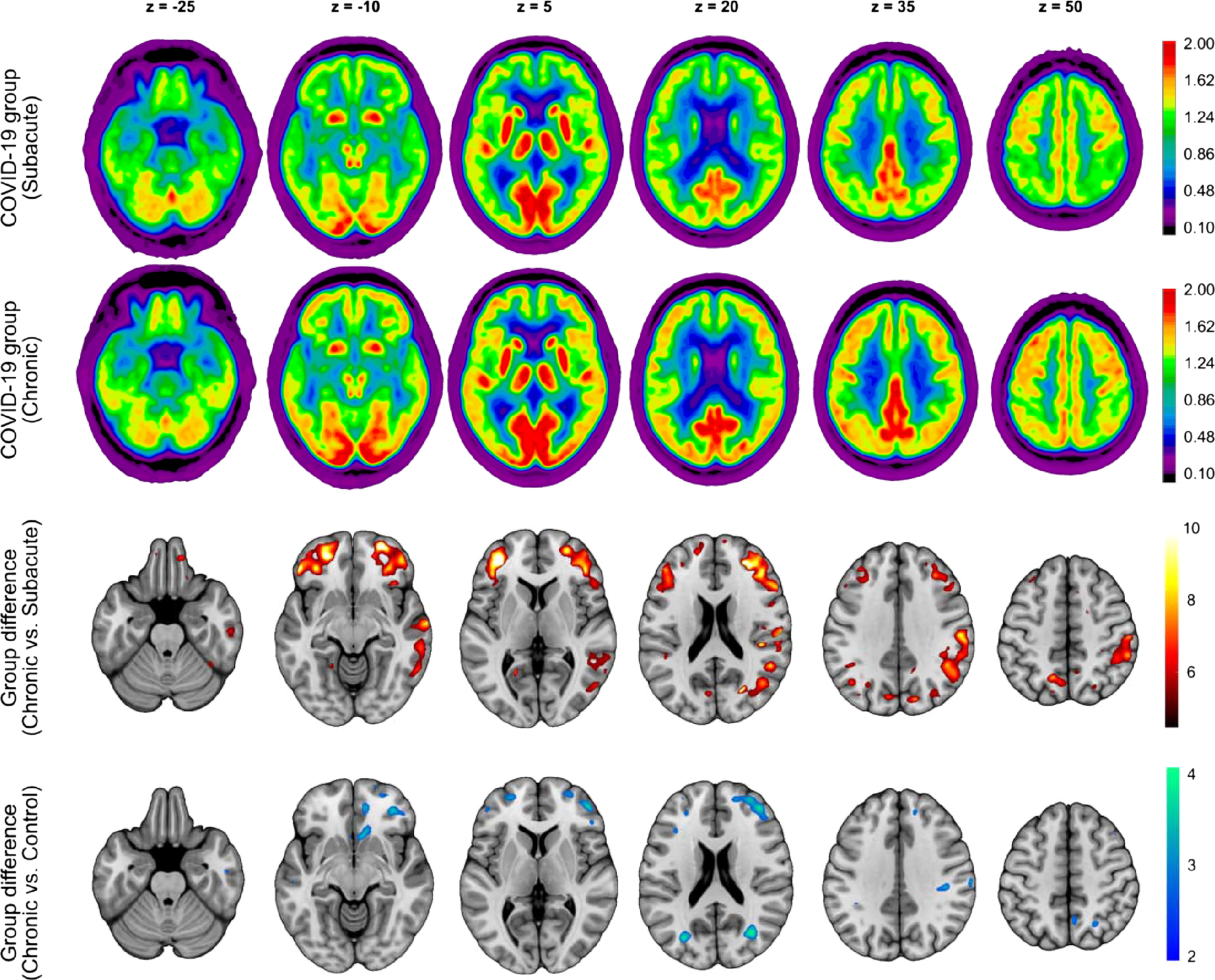
Result of ^18^F-FDG PET group analysis. (First and second rows) Transaxial sections of group-averaged, spatially normalized ^18^F-FDG PET scans in COVID-19 patients at subacute and chronic stages (*n* = 8; initially requiring inpatient treatment for nonneurologic complications). (Third and fourth rows) Results of statistical parametric mapping analysis. Third row illustrates regions that show significant increases in normalized ^18^F-FDG uptake in COVID-19 patients at chronic stage compared with subacute stage (paired *t* test, *P* < 0.01, false-discovery rate–corrected). Fourth row depicts regions that still show significant decreases in normalized ^18^F-FDG uptake in COVID-19 patients at chronic stage compared with age-matched control cohort (2-sample *t* test, *P* < 0.005). SPM12 *t* values are color-coded and overlaid onto MRI template. Images are presented in neurologic orientation, that is, left image side corresponds to patient’s left body side; numbers denote axial (*z*) position in millimeters

The averaged ^18^F-FDG PET images of the COVID-19 patients at both stages, as well as the regions that showed a significant increase in regional glucose metabolism at the chronic stage compared with the subacute stage (*P* = 0.01, false-discovery rate–corrected, corresponding to *t* > 5.31) are displayed in Figure 2. Group analysis using SPM12 revealed a widespread increase in ^18^F-FDG uptake in the frontoparietal and, to a lesser extent, temporal neocortical regions at the chronic stage compared with the subacute stage. No regions with a significant decrease in glucose metabolism were identified. Voxelwise comparison of chronic-stage patients to the age-matched control cohort confirmed the presence of a remaining neocortical hypometabolism in COVID-19 patients even at the chronic stage at an exploratory statistical threshold (*P* = 0.005 corresponding to *t* > 2.68; no significant cluster at *P* = 0.01, false-discovery rate–corrected; Fig. 2).

## DISCUSSION

In the present follow-up study, we demonstrated essential reversibility of decreased neocortical glucose metabolism assessed by ^18^F-FDG PET accompanied by an improvement in cognitive functions in COVID-19 patients from the subacute stage to the chronic stage after a SARS-CoV-2 infection. The expression of the previously established COVID-19–related spatial covariance pattern at the chronic stage was significantly reduced compared with what it was at the subacute stage. However, in comparison to a control cohort, chronic COVID-19 patients still exhibited a slightly higher pattern expression (at trend level) and residual hypometabolism indicating a shift toward normal levels, but no definite return. Although we observed a significant improvement in the cognitive screening test (MoCA), the average performance was still within the range of mild cognitive impairment (*6*). This slow but evident recovery provides fundamental and novel insights into the pathophysiology of cognitive deficits associated with COVID-19.

Recent neuropathologic examinations of patients whose death from COVID-19 was due to nonneurologic causes shed light on potential pathophysiologic mechanisms underlying the sustained cortical hypometabolism and impairment (*12*): SARS-CoV-2 RNA or proteins could be detected in 53% of patients, with a predominance for the caudal brain stem and cranial nerve, highlighting the known neuroinvasive propensity of human β-coronavirus clades (*13,14*). However, major histopathologic findings were astrogliosis, microglial activation, and mild infiltration by cytotoxic T lymphocytes, with an emphasis on brain stem and cerebellum (*12*), that were unrelated to the presence of SARS-CoV-2. Therefore, these changes are more likely caused by a systemic inflammatory response or cytokine release (*15*). Because the cortical gray matter is largely spared from damage and inflammatory changes (*5,12*), the reduced glucose metabolism is likely secondary; for example, it may be a consequence of a functional decoupling from aminergic brain stem nuclei (*16,17*). This inflammation-triggered process could have outlasted the acute infection and only partly recovered over the contemplated period of 6 mo. Of note, changes in cognition (MoCA) seem to be associated with changes in PES, although this observation failed to attain statistical significance (*r* = −0.54, *P* = 0.16). We did not observe an association between changes in PES and MoCA on the one hand and time to follow-up examinations on the other. This lack of association may also be due to the limited number of subjects and the relatively narrow and late time range of follow-up examinations. Still, the regions with residual hypometabolism are those with the most prominent decreases during the acute stage and may thus take longer to fully recover. Consequently, the slow reversibility of post–COVID-19 cerebral hypometabolism and cognitive impairment described in the present study would be in accordance with a lasting perturbation of cortical function caused by a subcortical periinflammatory process as a correlate of the post–COVID-19 syndrome.

Comparison of the present study to other studies using ^18^F-FDG PET to assess COVID-19–associated metabolic deficits is hampered by various factors. For instance, Guedj et al. (*8*) reported a cohort of patients examined at highly variable time points (about 1–5 mo after COVID-19; on average, 96 ± 31 d). Given the apparent time dependency of cognitive and metabolic changes, such pooling of patients at presumably different stages precludes a comparison to studies of selected time points such as ours. Moreover, given the obvious alterations in cortical metabolism observed in our cohort and the study by Kas et al. (*7*), the use of cortical regions for count rate normalization of PET data appears problematic. In fact, this is why we selected an approach (i.e., principal-components analysis) that does not require an a priori definition of a reference region. Such factors (among others) might lead to discordant results and contraintuitive findings, such as an association between a decreasing glucose metabolism and a longer time after the first COVID-19 symptoms (*8*), as is contrary to both the study from Kas et al. (*7*) and our study. In turn, our study and that of Kas are in good agreement with each other, showing a slow, though not (yet) complete recovery of cortical metabolism over 6 mo. Still, a detailed comparison of these 2 studies is complicated by the initial inclusion criteria: patients were prospectively enrolled in our cohort when presenting with at least 1 new neurologic symptom and underwent ^18^F-FDG PET for further diagnostic work-up if at least 2 new symptoms were present (*5*). Kas et al. (*7*) reported the analysis of ^18^F-FDG PET scans in 7 patients who had COVID-19–related encephalopathy with new-onset cognitive impairment. Moreover, initial disease severity was different in the 2 studies (i.e., 3/7, or 43%, of patients reported by Kas et al. (*7*) needed mechanical ventilation, as opposed to 2/8, or 25%, in the present study). Finally, Kas et al. (*7*) also conducted an assessment by a comprehensive cognitive test battery, but no direct association to PET data was reported.

A limitation of the present study is the small sample size, with only 8 of 17 initial patients receiving a follow-up ^18^F-FDG PET and MoCA examination. Obviously, patients with actual cognitive complaints are more likely to adhere to a follow-up program than are subjectively healthy patients. In fact, 6 patients declined follow-up because they lacked self-perceived complaints, whereas only 4 of 8 patients denied cognitive impairments in our actual sample. However, this selection bias is inherently linked to the present study’s major finding of slow but evident recovery of cognitive impairment. In addition, the selection of the initial cohort (only inpatient—not dominant intensive-care-unit—treatment) limits the generalizability of our findings (especially to outpatients, representing most COVID-19 patients). Furthermore, there may be premorbid conditions or risk factors rendering subgroups of patients particularly susceptible to COVID-19–associated cognitive impairments. No obvious factors were identified in the present prospective single-center study (including the initial sample) (*5*). However, future larger, population-based studies are needed to address this question.

## CONCLUSION

Given the current pandemic situation and the still tremendous uncertainty concerning the long-term sequelae of COVID-19, the present study provides novel insights of the highest medical and socioeconomic relevance. We provide evidence of longer-lasting metabolic and accompanying cognitive deficits after COVID-19. Although a significant recovery of regional neuronal function and cognition can be clearly stated, residuals are still measurable in some patients 6 mo after manifestation of COVID-19. In consequence, post–COVID-19 patients with persistent cognitive complaints should see a neurologist and possibly enter cognitive rehabilitation programs.

## DISCLOSURE

Nils Schroeter was supported by the Berta-Ottenstein-Programme for Clinician Scientists, Faculty of Medicine, University of Freiburg. Andrea Dressing was supported by the Berta-Ottenstein Programme for Advanced Clinician Scientists, Faculty of Medicine, University of Freiburg. Philipp Meyer received honoraria from GE (presentation, consultancy) and Philips (presentation). No other potential conflict of interest relevant to this article was reported.

**KEY POINTS**

**QUESTION:** Are cognitive impairment and associated cortical hypometabolism reversible approximately 6 mo after COVID-19?
**PERTINENT FINDINGS:** In 8 patients with COVID-19–associated deficits in cognitive impairment and cortical hypometabolism at the subacute until the chronic stage, ^18^F-FDG PET revealed that the initial frontoparietal and, to a lesser extent, temporal glucose hypometabolism was reversible and that this reversibility was accompanied by a significant improvement in cognition. Expression of the previously established COVID-19–related covariance pattern was significantly lower at follow-up and correlated inversely with MoCA performance.
**IMPLICATIONS FOR PATIENT CARE:** Although significant recovery of regional neuronal function and cognition is clear, residuals are still measurable even 6 mo after manifestation of COVID-19. Post– COVID-19 patients with persistent cognitive complaints should see a neurologist and possibly enter cognitive rehabilitation programs.
